# Supplementary material for: Patterns of Intron Gain and Loss in Fungi
Source: PLoS Biol. 2004 Nov 30;2(12):e422. doi: 10.1371/journal.pbio.0020422 (PMC532390; doi:10.1371/journal.pbio.0020422)
Supplement: Table S1 — Also available at http://genes.mit.edu/NielsenEtAl/. (4.3 MB ZIP). [file pbio.0020422.st001.zip › NielsenEtAl/html/219.html]

AN2416.1.NCU02532.1.MG09283.1.FG00364.1


```
 CLUSTAL W (1.82) Multiple Sequence Alignments - Introns Inserted


Sequence 1: MG09283.1	378 aa
Sequence 2: FG00364.1	433 aa
Sequence 3: NCU02532.1	433 aa
Sequence 4: AN2416.1	382 aa
Alignment Length: 439 aa
Number Identitical Residues: 183 aa
Alignment Score (without introns) 9740


MG09283.1 	MAADTEMIAG-------~-----VDTSSQRC2FDPDS-----CVSSMEKMSVL2------
NCU02532.1	MSSPEVLPDS--EAKRW2KYLNNVRTRPGAF~VDPEEFDGSTVVENMNKIKIF2GAGGLG
FG00364.1 	MSCDVQSPAGGHQNTRW2KYLDQIRQNAGPY~TDPEAIA-PEALEGFNTLKVL2GAGGLG
AN2416.1  	MGAG----------NQW2KYLYEVLSKSGPF~SDPDWVPGNETIKSLEESKIL2------
          	*..           .   .   .:   .     **:   .   :..::  .::       

MG09283.1 	---------------------~---------~QFLFRKDDVGKYKAEVAARFVEKRVKDV
NCU02532.1	CEILKNLALSGFKDISVIDMD1TIDISNLNR~QFLFRHADVGKFKAEVAAKFVERRVKGV
FG00364.1 	CEILKNLAMSGFKNIHVIDMD1TIDISNLNR~QFLFRKDDVGKYKAEVAAAFVEKRVKGV
AN2416.1  	---------------------~-------VM2QFLFRQSDIGKPKAEVAAAFVERRVKGV
          	                                *****: *:** ****** ***:***.*

MG09283.1 	KITPHNCRIQEFDDDFYMQFQLVVCGLDSIEARRWINAKLCDMVDMD-NPDSFKPLIDGG
NCU02532.1	KITPYNCKIQDFDEDFYQQFQIVVCGLDSIEARRWINATLVNMASCE-NPDSMKPLIDGG
FG00364.1 	SITAHNNRIQDFDEEFYKQFQLVICGLDSIEARRWINAMLVSIAEEGEDADALKPLIDGG
AN2416.1  	KITPFVGKIQDKDEDYYMQFKIIVCGLDSIEARRWINSTLVGMVDID-NPESLKPLIDGG
          	.**..  :**: *:::* **::::*************: * .:..   :.:::*******

MG09283.1 	TEG~FKGQSRVIFPTMTACIECQLEIYAPRPAVPLCTIATIPRQPEHCIEWAHIIAWEKE
NCU02532.1	TEG~FKGQARVILPTMGSCIECQLDMHAPRAAVPLCTLASIPRQPEHCIEWAHVIAWDKE
FG00364.1 	TEG~FKGQARVILPTMTSCIECQLDMHAPRAAVPLCTIASIPRQPEHCVEWAHVIAWDKE
AN2416.1  	TEG1FKGQARVILPTLTSCIECQLDMHAPRPAVPLCTIATIPRQPQHCIEWAHQIAWQDK
          	*** ****:***:**: :******:::***.******:*:*****:**:**** ***:.:

MG09283.1 	KPFPALDKDDPEHITWLFQKAADRAKEYGIQGVTYSLTQGVVKNIIPAIASTNAIIAASC
NCU02532.1	KPFPQLDKDDPEHITWLYQKALERAKEFNISGVTYSLTQGVVKNIIPAIAATNSVIAAAC
FG00364.1 	KPFPKLDKDDPEHVTWLFQKALTRAQEFGIPGVTYSLTQGTIKNIIPAIASTNAIIAAAC
AN2416.1  	RKDDTFDSDDMEHIGWVYNAALERAKEFNISGVTFQMTQGVVKNIIPAIASTNAVIAAAT
          	:    :*.** **: *::: *  **:*:.* ***:.:***.:********:**::***: 

MG09283.1 	CNEAFKIASNAAPPLG----LEENYMMYTGDAGIYTFTYKYERKPDCPVCGNEARQLEVD
NCU02532.1	CNEALKIASSCAPFLGTPGEGEPNYMMYSGNDSIYTYTFKHEQKEDCPVCGMTARVLKVN
FG00364.1 	CNEAFKIATSSAPCLG----FQTNYMMYSGNDSIYTYTFKHEKKDDCPVCGRQARPLEVD
AN2416.1  	TSEALKIATSCNPFLE-------NYMMYAGEEGVYTYTFEAEKKPDCPVCGNLARKITVN
          	 .**:***:.. * *        *****:*: .:**:*:: *:* ******  ** : *:

MG09283.1 	PSWTLQELVDHLAVQPAAQLKKPSIRAEGKSLYVQMTPDLEKATRGNLTKTLTELGLVSG
NCU02532.1	PKWTLEELIESFATLPEAQLKKPSVRAEGKTLYMQLPPNLEEQTRPNLEKTLEQLGLTEG
FG00364.1 	PKTTLQELIESFAIRPEAQLKKASVRAEGKTLYMQFPPSLEEQTRPNLNKTLNELGLEDG
AN2416.1  	PDATLEEFIETLGERPEAQLKKPSLRTEEKTLYQRFPPQLEEQTRPHLKQKLRDL-IVSG
          	*. **:*::: :.  * *****.*:*:* *:** ::.*.**: ** :* :.* :* : .*

MG09283.1 	QEIAVTDPAFVSTVFQYRLQFK---
NCU02532.1	TELAVTDPAFVGVVFNFSLKFA---
FG00364.1 	QQVVVTDPAFP-LEFNFFFKFKTAS
AN2416.1  	QEVAVSDPAYT-IDFRFQLVFG---
          	 ::.*:***:    *.: : *
```
